# Landscape Effects on the Cabbage Seedpod Weevil, *Ceutorhynchus obstrictus* (Coleoptera: Curculionidae), and on Its Parasitoid, *Trichomalus perfectus* (Hymenoptera: Pteromalidae), in Canola

**DOI:** 10.3390/insects14040327

**Published:** 2023-03-28

**Authors:** Marie D’Ottavio, Sébastien Boquel, Geneviève Labrie, Eric Lucas

**Affiliations:** 1 Laboratoire de Lutte Biologique, Département des Sciences Biologiques, Université du Québec à Montréal (UQAM), Case Postale 8888, Succursale Centre-Ville, Montréal, QC H3C 3P8, Canada; 2 Centre de Recherche Sur Les Grains Inc. (CÉROM), 740, Chemin Trudeau, Saint-Mathieu-de-Beloeil, QC J3G 0E2, Canada; 3 Centre de Recherche Agroalimentaire de Mirabel (CRAM), 9850, Rue Belle-Rivière, Mirabel, QC J7N 2X8, Canada

**Keywords:** parasitism rate, biological control, agroecosystem, spatial context, Nearctic landscape, pest

## Abstract

**Simple Summary:**

The exotic cabbage seedpod weevil (CSW), *Ceutorhynchus obstrictus*, is a major pest of canola crops. This insect is mainly regulated in Europe by the parasitoid *Trichomalus perfectus*, a natural enemy also accidentally introduced in eastern Canada since 2009. The objective of the present study was to evaluate how the landscape influenced the CSW infestation and abundance and the parasitism of *T. perfectus*. Results from six years in eight Quebec regions show that the CSW was positively affected by roads and cereal crops. Regarding *T. perfectus*, the parasitism was variable (from about 5 to almost 25%) and positively influenced by landscape diversity, crop edge density, hay/pastures and soybean crops. These results will help determine the potential of an eventual introduction of *T. perfectus* in western Canada, where most of the canola is produced, and the optimal landscape composition and configuration needed to succeed.

**Abstract:**

The cabbage seedpod weevil (CSW), *Ceutorhynchus obstrictus*, an exotic pest accidentally introduced in North America in 1931, spread all over this continent and is now a major pest of canola crops. One of its main natural enemies in Europe, *Trichomalus perfectus*, was observed in eastern Canada in 2009. This study aimed to evaluate the landscape influence on CSW infestation and abundance and on *T. perfectus* parasitism in Quebec to understand the optimal conditions to potentially release this parasitoid in the Canadian Prairies. Field research was conducted in 19 to 28 canola fields per year, from 2015 to 2020, among eight Quebec regions. CSW was sampled by sweep net during canola blooming and parasitoids by collecting canola pods kept in emergence boxes until adults emerge. Infestation and parasitism calculations were based on pod emergence holes. For analysis, 20 landscape predictors were considered. Results show that CSW infestation and abundance increased if there were more roads and cereal crops in the landscapes. Meanwhile, *T. perfectus* parasitism decreased when hedgerows length and distance from water were longer. However, it increased when landscape diversity and average crop perimeter-to-area ratio were higher, and along with more hay/pastures and soybean crops. This study’s results highlight that these four landscape predictors could provide more resources and overwintering areas, promoting greater efficiency of *T. perfectus* to control the CSW.

## 1. Introduction

Canola, *Brassica napus* L. (Brassicaceae), an oilseed plant genetically derived from the oilseed rape, is largely cultivated in North America [1] and more specifically in the Canadian Prairies (Alberta, Saskatchewan and Manitoba) [2]. Canada and Europe are the main producers with respectively 19.49 million metric tons of canola (26.64%) and 16.29 million metrics tons of oilseed rape (22.27%) of world production (2020–2021) [3]. The cabbage seedpod weevil (CSW) *Ceutorhynchus obstrictus* (Marsham) (Coleoptera, Curculionidae), a palearctic species and a major pest of canola and oilseed rape crops [4,5,6,7], was accidentally introduced in 1931 in North America (Vancouver, Canada) [8,9] and spread eastward. In 1995, it was first observed in the Canadian Prairies and by the early 2000s in Ontario and southern Quebec (Canada) [9,10,11]. Mating occurs during canola blooming and females feed on buds, flowers, and pods before laying eggs into pods [12]. Larvae cause the worst damage by feeding on pod seeds during two to three weeks and can reduce seed weight by 20.2% per pod [6]. Third instars chew a hole in the pod wall and fall to the ground to pupate [4,5]. The main control of this pest is achieved by foliar insecticides (pyrethroids) during canola bloom [13,14], when the threshold of 3–4 CSW per sweep is reached [7].

In several European countries, a survey of CSW parasitoids has been carried out [14,15,16,17,18]. In the *B. napus* crop, the main CSW parasitoid is *Trichomalus perfectus* (Walker) (Hymenoptera, Pteromalidae), an oligophagous univoltine ecto-parasitoid of *Ceutorhynchus* spp. larvae [18], which detects the CSW by feces scents [14,19,20]. A high parasitism rate can be observed, even when CSW density is low [21,22]. At maturity, the adult parasitoid chews a hole to exit the pod and mates before the female overwinters [14,19]. In Germany, *T. perfectus* represents 89% of the CSW parasitoid community [18]. Overall parasitism rates are 35% in Romania, 27% in Switzerland and 21% in Germany; *T. perfectus* parasitism rates in those three countries are between 6.52 and 19.10% and on average are 12.46% [18]. In Estonia, the *T. perfectus* parasitism rate is about 61% of the overall parasitism rate [17]. In Canada, the presence of *T. perfectus* parasitizing the CSW was confirmed in 2009 in Quebec and Ontario. Out of 10 individuals collected from *B. napus*, 40% were *T. perfectus* in Southern Quebec, and then out of 16 individuals, 46.7% were *T. perfectus* in Ottawa (Ontario) [23]. The average overall parasitism rate in Quebec from 2012 to 2020 was 26.7% [24], unpublished data. 

Currently, only two studies analyzed the effects of landscape predictors on the CSW in Estonia [25,26]. Abandoned fields, hayfields, wheat crops and permanent grasslands were landscape composition elements that increased CSW infestation rate. Herbaceous linear bordering oilseed rape crops was a landscape configuration element that also increased the CSW infestation rate [26]. However, Kovács et al. [25] found no significant effect of the habitat bordering the oilseed rape crop. Kovács et al. [25,26] considered overall parasitism rates (all parasitoid species) to analyze landscape composition and configuration effects. Permanent grasslands and roads decreased overall parasitism rates, but more herbaceous linear elements next to the oilseed rape crop increased overall parasitism rates [26]. Moreover, no significant effect of the landscape elements adjacent to the oilseed rape crop or the distance from the field edge (from 2 to 75 m) was found on overall parasitism rates. However, a lower parasitism rate was observed in oilseed rape crops adjacent to woody elements [25,26]. To date, a single study analyzed landscape configuration effect on *T. perfectus* abundance in Sweden, showing that the parasitoid was mainly present in oilseed rape crops not very close to forests, i.e., more than 2 km [27]. 

The objectives of the present study were to evaluate landscape effects on the CSW and on the parasitism rate of its main parasitoid *T. perfectus* in Quebec. It will also help to understand the optimal conditions needed to potentially release this parasitoid in the Canadian Prairies to control the CSW. This is the first study considering effects of landscape predictors on these insects in North America and considering *T. perfectus* parasitism rate alone. 

The first hypothesis was that higher availabilities of canola crops, hay/pastures and forests in the landscape would increase CSW abundance and infestation rate. If the landscape is dominated by the host crop, a pest has a higher probability to find it because of a high nutritional resource concentration [28,29,30]. Moreover, hayfields increased CSW infestation rate [26], and the CSW would overwinter under soil or litter of woodlands [5,31,32]. The second hypothesis was that higher proportions of main crops (corn, wheat, soybean) in the landscape would decrease the *T. perfectus* parasitism rate. Crops are not stable habitats (tillage, rotation) [33], and specialist parasitoids were more sensitive to the presence of several crops compared to their hosts [34]. The third hypothesis was that higher field edge density and hedgerow length in the landscape would increase the *T. perfectus* parasitism rate. Floral resources in these habitats promoted parasitoid longevity and fecundity [35,36]. Furthermore, field edges led to high CSW parasitism rates [25,26]. The fourth hypothesis was that a higher distance between the canola crop and a semi-natural source habitat and a higher number of patches would decrease the *T. perfectus* parasitism rate. Biocontrol by parasitoids was higher near their source habitat [37,38], and small female parasitoids can spread less than big ones [39]. Furthermore, fragmentation led to the lost of specialist parasitoids [40]. 

## 2. Materials and Methods

### 2.1. Sampling Methods: Sweep Nets for the CSW and Pod Collection for T. perfectus

**Figure 1:**
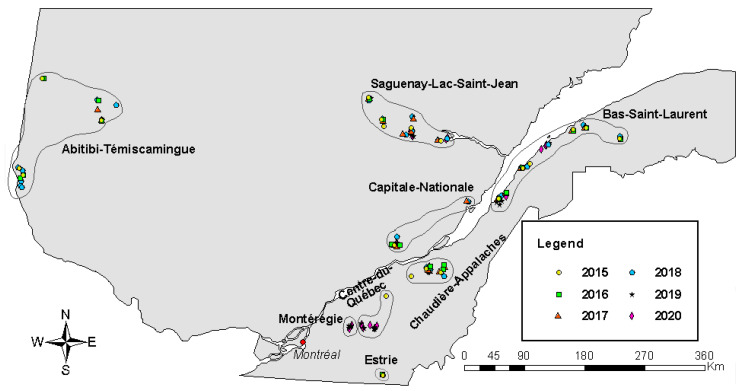
Distribution of the 140 sampled canola fields in eight Quebec regions (from 47°25′44.4″ N, 79°27′42.1″ W to 48°24′14.5″ N, 67°24′9.9″ W), from 2015 to 2020.

Data were collected during the summers of 2015 to 2020. From 19 to 28 canola fields were sampled each year for a total of 140. During the six years, the same canola field was never sampled twice, except one field in 2019 and 2020. In total, eight different regions where canola is produced were considered for the sampling: Montérégie (2019, 2020), Estrie (2015, 2016), Centre-du-Québec (2015, 2019, 2020), Chaudière-Appalaches (2015, 2016, 2017, 2018, 2019, 2020), Capitale-Nationale (2015, 2016, 2017, 2018, 2019, 2020), Bas-Saint-Laurent (2015, 2016, 2017, 2018, 2019, 2020), Saguenay-Lac-Saint-Jean (2015, 2016, 2017, 2018, 2019, 2020) and Abitibi-Témiscamingue (2015, 2016, 2017, 2018) (Figure 1).

The CSW abundance was evaluated once during flowering peak period in July using a sweep net (38 cm diameter). For each field, 10 standard 180° arc sweeps were performed while walking in a straight line in five (2015 to 2018) to 10 (2019 and 2020) stations. After each sampling (a total of 10), individuals were counted in the field and released, and then mean abundance was calculated for each canola field. The CSW was clearly the most abundant Ceutorhynchinae species and easily discernable since it is bigger than other Ceutorhynchinae species that may be present in canola fields. At maturity, a total of 1000 pods that had reached the final size were collected in each canola field close to the edges (up to 20 m in the canola field). 

### 2.2. Emergence Boxes (CSW and Parasitoids)

From 2015 to 2018, 1000 pods were collected per field and placed in an emergence box (30 × 20 × 18 cm), while from 2019 to 2020, 1000 pods from the same field were distributed in four emergence boxes (250 pods per box) but grouped for calculations. All emergence boxes were fully sealed but had a transparent plastic tube stuck into a hole on one side of the box, allowing light penetration to attract emerging parasitoids [41]. Live parasitoids were collected for identification [42] and rearing. Approximately four weeks later, when no more living parasitoid was found, boxes were opened and emergence holes (of CSW and/or of parasitoids) on pods, dead emerged CSW larvae and dead emerged parasitoids were counted. Each pod was carefully inspected for holes and *T. perfectus* was identified following the identification key of Muller et al. [42] using a stereomicroscope (LEICA MZ6, Wetzlar, Germany). Parasitism and infestation rates were calculated for each canola field. Overall and *T. perfectus* parasitism rates were calculated considering respectively the number of all emerged parasitoids and emerged *T. perfectus*, because the distinction between the holes of parasitoids versus CSW was not always obvious. Moreover, it was impossible to differentiate emergence holes between different parasitoid species. 

The infestation and parasitism rates formulas used are as follows (where *n* represents the upper limit of summation and *i* represents the index of summation):

$$\mathrm{CSW}\;\mathrm{infestation}\;\mathrm{rate}:\;\left(\frac{\sum_{i}^{n}pods\;with\;emergence\;holes\;in\;a\;box}{\sum_{i}^{n}pods\;in\;a\;box}\right)\times 100$$

$$\mathrm{Overall}\;\mathrm{parasitism}\;\mathrm{rate}:\;\left(\frac{\sum_{i}^{n}emerged\;parasitoids\;in\;a\;box}{\sum_{i}^{n}pods\;with\;emergence\;holes\;in\;a\;box}\right)\times 100$$

$$T.\;\mathrm{perfectus}\;\mathrm{parasitism}\;\mathrm{rate}:\;\left(\frac{\sum_{i}^{n}emerged\;T.\;perfectus\;in\;a\;box}{\sum_{i}^{n}pods\;with\;emergence\;holes\;in\;a\;box}\right)\times 100$$

### 2.3. Landscape Predictors Measures 

All field GPS coordinates were first compiled and then landscape predictor were measured using ArcGIS software 10.6 (ESRI, Redlands, CA, USA, 2017) according to the NAD83/Quebec Albers coordinate system. Measures were based on two shapefiles: One associated with the crops (Financière Agricole du Québec, Gouvernement du Québec, Canada) and the other associated with all landscape categories (Utilisation du territoire, Gouvernement du Québec, Canada). Hedgerow lengths were based on the ArcGIS basemap World Imagery. Measures were centered from each sampled canola field by considering a 1 km diameter around it, knowing that the whole field was considered in each landscape. A larger spatial scale was not considered so as to avoid landscape overlap and consequently data loss. Furthermore, this is the most common scale used in landscape studies, especially with parasitoids, and landscape data were more reliable at this scale in several regions. A total of 30 landscape predictors were measured but 20 were tested in the analysis (Table 1). All area measures were then converted into proportions compared to the total buffer area considered.

### 2.4. Statistical Analysis

All statistical analyses were performed with R software (V. 4.0.3) [43] using the “lme4”, “car”, “MASS” and “MuMIn” packages. A correlation matrix allowed to check for potential correlations between landscape variables with a threshold of 75% (Spearman coefficient). If two variables had a correlation higher than 75%, the one with more biological meaning was considered in following analysis. Some landscape variables were not considered because of a lack of biological meaning and/or due to having very few data points.

*Linear Mixed Models (LMMs)* were used for two dependent variables: CSW infestation rate and CSW abundance. To achieve normality of residuals and homoscedasticity, a log transformation was applied for CSW abundance. Additionally, since CSW infestation rate variable was made up of proportions and had several values outside the 0.3–0.7 range and equal to 0, an arcsine-square-root transformation was applied [44]. A *Generalized Linear Mixed Model (GLMM)* with a binomial distribution was used for the *T. perfectus* parasitism rate. For each model, a threshold of α = 0.05 was fixed, region and year were included as crossed random effects and a stepwise regression (bidirectional) was used. The independent variable CSW abundance was tested in the model considering the *T. perfectus* parasitism rate as a dependant variable. A “*weight*” parameter was added to the CSW infestation rate model and the “*cbind*” function was used for the *T. perfectus* parasitism rate model. Indeed, the total number of pods could slightly differ between emergence boxes (counting bias) and some *T. perfectus* parasitism rate values were very high while there were very few emergence holes. Potential multicollinearities were checked between independent variables with the *Variance Inflation Factor (VIF)* function. In case independent variables produced multicollinearity, they were dropped from the models. Finally, the best models were chosen according to the lowest second-order *Akaike Information Criterion (AICc)* value when comparing models after adding or dropping a variable, with the *Maximum Likelihood (ML)* approach. *AICc* was chosen due to the small sample size (n/k < 40) [45]. Final models were considered with the *Restricted Maximum Likelihood (REML)* approach for linear mixed models also due to the small sample size. 

Ultimately, three correlation tests were performed using the Kendall coefficient: (i) CSW abundance versus CSW infestation rate; (ii) CSW infestation rate versus *T. perfectus* parasitism rate; (iii) CSW abundance versus *T. perfectus* parasitism rate.

## 3. Results

### 3.1. Overall Situation

**Figure 2:**
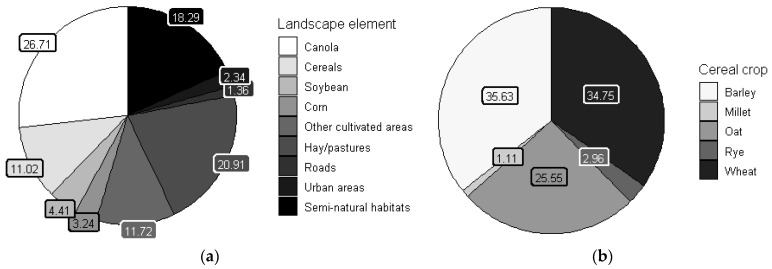
(**a**) Mean proportions (%) of landscape elements per landscape combining all years and regions. Undifferentiated crops (8.21%), mixed crops (1.65%) and other crops (1.86%) are included in other cultivated areas. Forests (14.76%), aquatic areas (1.23%), shrublands (1.48%) and bogs (0.82%) are included in semi-natural habitats; (**b**) mean proportions (%) of cereal crops per landscape combining all years and regions.

Considering all years and regions, on average, landscapes were dominated by canola crops (26.71%) and hay/pastures (20.91%). Corn was less cultivated (3.24%) than other major crops (soybean but mainly canola and cereals), and roads were less present (1.36%) than other landscape elements (Figure 2a). Proportion of cereals were mostly represented by barley (35.63%) and wheat (34.75%) crops, while oat crops came third (25.55%) (Figure 2b).

CSW abundance was highest in 2019 (9.24), and lowest in 2015 (1.80) and 2018 (1.09). CSW infestation rate was also highest in 2019 (6.91%) and lowest in 2015 (0.32%) and 2018 (0.86%). Moreover, the proportion of canola fields above the damage threshold was highest in 2019 (39.29%) and lowest in 2015 (4.76%). Meanwhile, overall and *T. perfectus* parasitism rates were highest in 2018 (23.09 and 14.93%, respectively), 2020 (26.82 and 21.76%, respectively), and especially 2015 (48.39 and 24.76%, respectively). Additionally, more parasitoids (14.00) and *T. perfectus* (10.72) emerged from 1000 pods on average in 2020, but less parasitoids (2.29) and *T. perfectus* (1.71) emerged on average in 2015. In particular, 2015 was a year with some high cases of parasitism rates but almost no emergence holes. The parasitism rate of *T. perfectus* was lowest in 2016 (5.40%) (Table 2).

### 3.2. CSW Abundance and Infestation 

**Figure 3:**
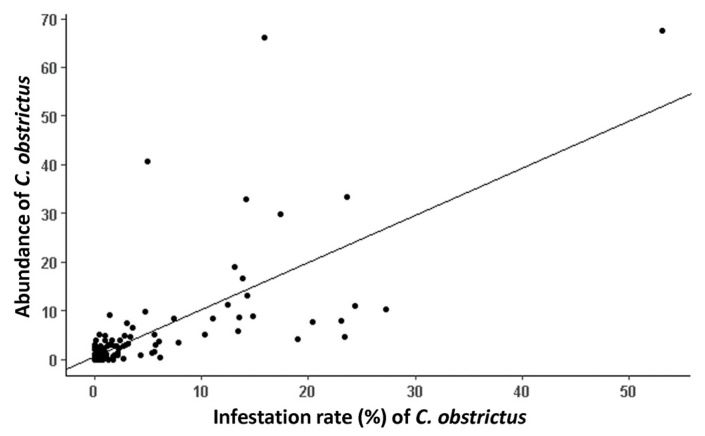
Relation between CSW abundance (average number) and CSW infestation rate (mean in %).

**Figure 4:**
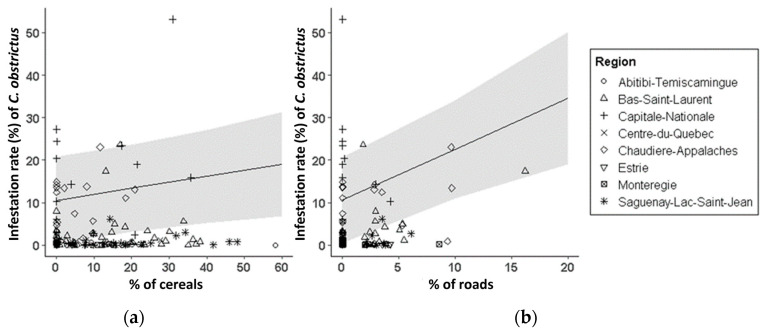
Landscape predictors: (**a**) % of cereals and (**b**) % of roads, with significant effects on CSW infestation rate from 2015 to 2020 in eight Quebec regions. Gray areas represent 95% confidence intervals (CI).

A significant positive correlation between CSW abundance (sweep nets) and CSW infestation rate (pods) was observed (*Kendall’s Tau* = 0.60, *z* = 9.79, *p* < 0.0001) (Figure 3). There were significant positive effects of roads (*t* = 3.69, *df* = 125.62, *p* < 0.001) and cereal crops (*t* = 2.05, *df* = 126.98, *p* = 0.041) on CSW infestation rate in canola crops (Figure 4, Table S1). CSW infestation rate was higher in canola crops when roads and cereal crops were more present in the landscape. Overall, Capitale-Nationale, Bas-Saint-Laurent and Chaudière-Appalaches regions showed the highest CSW infestation rates (Figure 4). There were also significant positive effects of roads (*t* = 3.26, *df* = 122.49, *p* = 0.001) and cereal crops (*t* = 2.09, *df* = 125.37, *p* < 0.039) on CSW abundance (Table S1).

### 3.3. Trichomalus perfectus Parasitism Rate 

**Figure 5:**
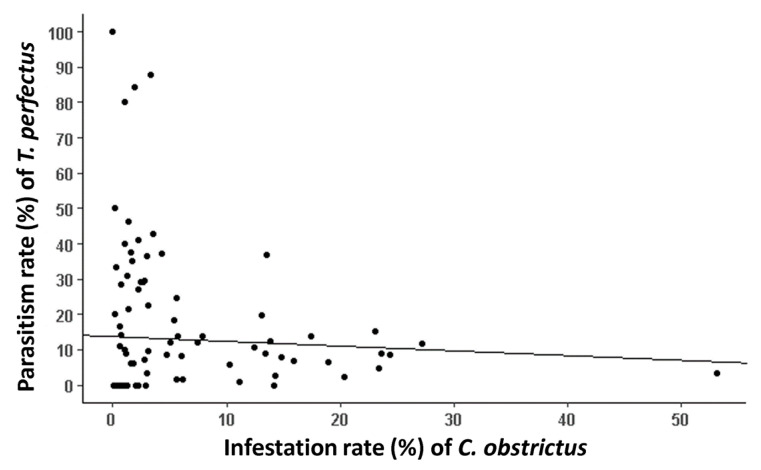
Relation between *T. perfectus* parasitism rate (mean in %) and CSW infestation rate (mean in %).

**Figure 6:**
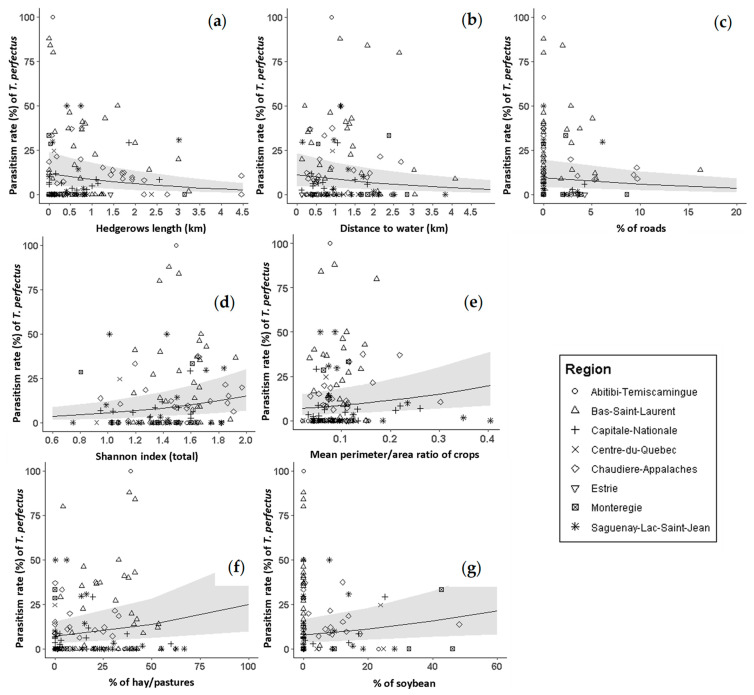
Landscape predictors: (**a**) Hedgerows length; (**b**) distance to water; (**c**) % of roads; (**d**) Shannon index (total); (**e**) mean perimeter/area ratio of crops; (**f**) % of hay/pastures; (**g**) % of soybean, with significant effects on *T. perfectus* parasitism rate from 2015 to 2020 in eight Quebec regions. Gray areas represent 95% confidence intervals (CI).

A highly significant but low negative correlation between *T. perfectus* parasitism rate and CSW infestation rate was observed (*Kendall’s Tau* = 0.29, *z* = 4.31, *p* < 0.0001) (Figure 5). A significant but lower negative correlation between *T. perfectus* parasitism rate and CSW abundance was observed (*Kendall’s Tau* = 0.21, *z* = 2.99, *p* < 0.003) (Figure S1). *T. perfectus* parasitism rate was lower in canola crops when more hedgerows were present in the landscape (*z* = −5.70, *SE* = 0.01, *p* < 0.001), and when the distance from the sampled canola field to the nearest aquatic area was longer (*z* = −3.31, *SE* = 0.01, *p* = 0.001). Additionally, the more roads in the landscape (*z* = −3.44, *SE* = 1.52, *p* = 0.001), the lower *T. perfectus* parasitism rate (Figure 6, Table S2). The total landscape Shannon index (*z* = 4.64, *SE* = 0.24, *p* < 0.001), the average perimeter-to-area ratio of crops (*z* = 3.71, *SE* = 0.84, *p* < 0.001), hay/pastures (*z* = 3.23, *SE* = 0.46, *p* = 0.001) and soybean crops (*z* = 2.66, *SE* = 0.72, *p* = 0.008) had positive significant effects on *T. perfectus* parasitism rate (Figure 6, Table S2). The more diverse the landscape and the higher the average perimeter-to-area ratio of crops, the higher *T. perfectus* parasitism rate. Moreover, the more soybean crops in the landscape, the higher *T. perfectus* parasitism rate. Overall, Bas-Saint-Laurent region showed the highest *T. perfectus* parasitism rates (Figure 6). 

## 4. Discussion

Regarding the CSW, the hypothesis that canola crops, hay/pastures, and forests in the landscape increase its infestation rate in canola crops was not supported. Indeed, these predictors had no effect on CSW infestation rate, but roads and cereals did have one. Regarding *T. perfectus*, the hypothesis that higher field edge density and hedgerow length in the landscape would increase its parasitism rate was partially validated. Indeed, a high field edge density promoted its parasitism rate, but the opposite effect was found for long hedgerow length since it did not promote *T. perfectus* parasitism rate. The opposite was also found regarding the hypothesis that higher proportions of main crops would decrease the parasitism rate, but only for one major crop. Soybean crop positively influenced *T. perfectus* parasitism rate. Finally, the hypothesis that a higher distance between the canola crop and a semi-natural source habitat would decrease *T. perfectus* parasitism rate was supported. Aquatic areas far from canola fields in fact did not promote *T. perfectus* parasitism rate. Patches in landscapes, nevertheless, had no effect. 

### 4.1. Landscape Effects on CSW Infestation and Abundance

Roads had a positive effect on both CSW infestation rate and abundance. Almost a third of landscapes (31.43%) contained roads, with an average of 4.03% roads in those landscapes, and a maximum of 16.20%. Roads do not act as barriers for continuous flight of CSW, in contrast to small and flightless insects [46]. CSW is an active flyer able to travel several kilometers in one season, and could be carried out by wind turbulence and convection movements at great altitudes [47]. During hot weather (25 °C), CSW was caught at a height of more than 3 m, and some catches were at a height of 6 m [47]. Therefore, it would benefit from open spaces of roads to spread in the landscape. Moreover, road edges may contain floral cruciferous and wildflowers resources on which CSW could feed or lay eggs in pods [48]. Studies found that CSW feed on cruciferous species [9,12,48,49,50]. Desroches et al. [48] showed that CSW adults were largely present during 2019 and 2020 on *Barbarea vulgaris* W. T. Aiton (Brassiccaceae) and *Raphanus raphanistrum* L. (Brassicaceae) present in canola edges, crop edges and road edges near canola crops. They also found CSW larvae in *Brassica campestris* L. (Brassicaceae), *Erysimum cheiranthoides* L. (Brassicaceae) and *R. raphanistrum* pods. Finally, several studies demonstrated that highway or road edges could be shelters or habitat corridors for arthropod species [51,52,53,54]. These corridors increase the global habitat area, enhancing the opportunities for target species to feed, reproduce [55] and act as overwintering sites for Curculionidae [56]. This could explain the positive effect of roads on CSW infestation rate observed in this study. 

Cereal crops also had a positive effect on both CSW infestation rate and abundance. This is in accordance with previous results from Kovács et al. [26], who found that wheat had a positive effect on CSW infestation rate in oilseed rape crops in Estonia. This positive effect can be explained by the crop rotation—when a canola crop is replaced by a cereal crop the next year. Indeed, in this study, out of 93 landscapes presenting at least one cereal crop, 43.01% presented at least one cereal crop in rotation with a canola crop. Moreover, a canola crop is regularly followed by a cereal crop in Quebec [57], especially barley (on average 35.63% of the landscape) and wheat (on average 34.75% of the landscape) in this study. At the end of the canola season, emerged CSW therefore seek floral resources near the canola field, before overwintering in the area [7]. Subsequently, these overwintering adults emerge in spring near the canola field that is now a cereal field. Furthermore, volunteer canola could also be present in the cereal crop, allowing for some CSW to stay, feed and lay eggs in pods. Whaley et al. [58] found that volunteer winter canola in winter wheat can often be a host to CSW in Douglas County (Washington, DC, USA). 

### 4.2. Landscape Effects on T. perfectus Parasitism Rate

*Trichomalus perfectus* parasitism rate did not increase if there were more CSW, probably because the host density availability was too high in canola fields. Apart from that, a negative effect of roads was observed on *T. perfectus* parasitism rate. Kovács et al. [26] also found a negative effect of roads on the overall parasitism rate, where *T. perfectus* accounted for 73.94% of all parasitoids. Some studies found a negative impact of traffic roads on insects in Canada (Ontario) [59,60], especially on Hymenoptera [59]. Insect abundance is lower when there is a high-traffic road [60], and roads are barriers, especially for small insects [46,61]. Therefore, *T. perfectus* could be more easily disturbed than CSW by traffic roads. Conventional mowing of roadsides could also have an impact since it leads to a 55% loss of Hymenoptera [62], which find fewer habitats. 

The positive effect of the perimeter-to-area ratio of crops on *T. perfectus* parasitism rate was related to edge density: the higher the ratio, the higher the crop edge density. Moreover, although some studies showed a positive effect of hedgerows on parasitoids [35,36,63], allowing individuals to find shelters and resources, the opposite was found in our study. These results are consistent with the fact that *T. perfectus* is probably not as a good flyer as other Hymenoptera because of its small size, between 1 and 3 mm [42]. Indeed, small female parasitoids disperse less than big ones [39]. 

On the one hand, too many hedgerows probably constitute barriers for the flight of *T. perfectus*, as is the case for other insects [64]. The parasitoid could be slowed down or stopped by hedgerows before reaching the canola crop. Additionally, Berger et al. [27] found that *T. perfectus* was mainly present in oilseed rape crops that were not close to forests (beyond 2 km). Trees probably act as physical barriers to *T. perfectus* flight. However, no distinction was made between hedgerow heights since this study relied on aerial photographs. Precise measurements would be required during fieldwork to further assess the maximum height before the parasitoid is significantly slowed down. 

On the other hand, after *T. perfectus* emerged from canola pods, females could look for floral resources in crop edges to store sugar or lipids before overwintering there. Previous studies showed the importance of floral edges. Indeed, foraging parasitoids use floral resources to increase sugar storage [65,66,67], especially females [68]. In a recent study, *T. perfectus* was the second-most abundant species (11.6%) in unmanaged and sowed habitats with *Onobrychis viciifolia* Scop. (Fabaceae) (included in oilseed rape crops). Additionally, during the ripening stage of oilseed rape crops, the parasitoid abundance was higher in habitats than in crops. Before *T. perfectus* overwinters, it probably looks for floral resources in these habitats, provided by dominating flowering plants such as *Tripleurospermum inodorum* L. (Asteraceae), *Lactuca serriola* L. (Asteraceae) and *O. viciifolia* [69]. The latter is frequently used as fodder and in flower strips to attract beneficial insects in agroecosystems [70]. *Trichomalus perfectus* could feed on these plant species, also present in Quebec [71], in crop edges before overwintering in the vicinity. It is moreover likely that *T. perfectus* prefers feeding on flowers with open nectaries, such as the cruciferous *Sinapis alba* L. (Brassicaceae) [72], in crop edges.

A higher *T. perfectus* parasitism rate when an aquatic area was close to the sampled canola field could indicate a microclimatic effect on this parasitoid, requiring a wetter environment. Because of high temperatures during summer, there is a greater risk of water loss through transpiration. This is especially the case for small insects that generally lose more water than big ones due to a larger relative surface area per body weight [73]. Some parasitoids therefore showed preference for high relative humidity (RH, 87%) [74], keeping their water content at an optimum level during summer [75]. High temperatures under wet [75,76] or relatively wet (60%) [77] conditions also promoted parasitism rates. Whereas some parasitoids reacted differently to low RH: oviposition and emergence of *Telenomus isis* (Polaszek) (Hymenoptera, Scelionidae) were both reduced [78], but other parasitoids generated higher parasitism rates at low RH [79,80]. 

A higher *T. perfectus* parasitism rate when the landscape was more diversified (more complex) suggests that this parasitoid could find diversified resources and shelters in semi-natural habitats. For instance, it could benefit from the microclimatic effect produced by aquatic areas, and the shade provided by trees/shrubs of forests and shrublands when the temperature is too high. Many studies found that more complex landscapes with more semi-natural habitats had a positive effect on natural enemies’ activities and abundance, including parasitoids [81,82,83]. Additionally, a more diversified landscape contains different crops. There consequently may be a higher probability that some crops have been canola the previous year (crop rotation), allowing *T. perfectus* to overwinter in the vicinity, and be already present in the landscape in the next year.

Finally, regarding crops, the positive effect of hay/pastures suggests a higher probability for *T. perfectus* to find nectar resources, in alfalfa and clover crops for instance. Alfalfa can increase the number of parasitoids [84], and Pteromalidae were associated with perennial flowers in alfalfa crops [85]. The positive effect of soybean crops on *T. perfectus* parasitism rate cannot be related to crop rotation nor to the presence of volunteer canola. A single soybean field was indeed a canola field the previous year. One hypothesis is that the parasitoid feeds on honeydew of the soybean aphid *Aphis glycines* (Matsumura) (Hemiptera, Aphididae) [68,86], bringing it an additional sugar resource. This aphid is a major pest in Quebec soybean crops [87]. Honeydew excreted on leaves by aphids may promote longevity and fecundity of Pteromalidae species [88]. Even parasitoids that do not parasitize aphids can feed on *A. glycines* honeydew, enhancing their nutrient storage [68]. Further assessments and observations would be relevant to better understand this effect.

## 5. Conclusions

In conclusion, on the one hand, landscapes with less or no roads and less or no cereal crops would not promote CSW infestation rate nor abundance. On the other hand, more complex landscapes, high crop edge density, presence of hay/pastures and soybean crops promote *T. perfectus* parasitism rate. The CSW impact in Quebec and Canadian Prairies canola crops should thus be reduced in landscapes (i) with as little roads and cereals crops as possible, (ii) with more complex landscapes, (iii) with a high crop edge density, (iv) and including hay/pastures and soybean in crop rotation. Other studies must be carried out to complete the portrait of the efficacy of *T. perfectus* parasitism on the CSW—for instance, by maximizing headcount prior to releasing *T. perfectus*, evaluating the impact of agronomical practices (e.g., pesticides, fertilizers, sowing dates) on *T. perfectus* and investigating its cold hardiness. These would be important steps in improving the success of this biological control agent.

## Figures and Tables

**Table 1:** Detailed information about landscape predictors measured. Numbers within brackets represent the number of landscape predictors measured and listed in the details column. Underlined predictors are those tested in the statistical analysis. Grey background is to highlight/differentiate more easily the two different kind of columns “Landscape Predictor” related to “Details”.

Landscape Predictor	Details	Landscape Predictor	Details
Area of each crop group (m^2^) (8)	Canola, cereals, corn, soybean, hay/pastures, other crops, mixed crops, and undifferentiated crops	Total landscape Shannon index (landscape diversity) (1)	$-\overset{s}{\underset{i=1}{\sum }}pi\;x\ln\left(pi\right)$ ^1^
Area of each semi-natural habitat (m^2^) (4)	Forests, aquatic areas (lakes, ponds, rivers…), shrublands, bogs	Crop Shannon index (crop diversity) (1)	$-\sum_{i=1}^{s}pi\;x\;\ln\left(pi\right)$ ^1^
Main roads and urban areas (m^2^) (2)	Urban areas: group of buildings, shops, golf	Number of semi-natural habitats patches, number of crop patches and total number of patches (3)	Distributed across all the landscape
Perimeter (m), area (m^2^) and perimeter-to-area ratio of sampled canola fields (3)	Related to size and field border density of sampled canola field	Distance between the sampled canola field and the nearest forest (m) (1)	Measured from the center of the sampled canola field
Average perimeter-to-area ratio of all crops (2)	Field border density index	Distance between the sampled canola field and the nearest aquatic area (m) (1)	Measured from the center of the sampled canola field
Landscape richness (1)	Number of different elements	Hedgerows length (m) (1)	Lines of trees/shrubs across all the landscape
Total crop area (m^2^) (1)	Eight crop groups considered altogether	Total semi-natural area (m^2^) (1)	Four semi-natural habitats considered altogether

^1^ *S* represents the upper limit of summation, *i* represents the index of summation, and *pi* represents the proportion of each landscape element (top formula) or the proportion of each crop (bottom formula).

**Table 2:** Summary of abundances, infestation and parasitism rates of pests and parasitoids from 2015 to 2020 combining all regions.

Year	Canola Fields Number	CSW Abundance (Mean per 10 Sweeps) (±SE)	CSW Infestation Rate (Mean in %) (±SE)	Parasitoids Number (Mean per 1000 Pods) (±SE)	*T. perfectus* Number (Mean per 1000 Pods) (±SE)	Overall Parasitism Rate (Mean in %) (±SE)	*T. perfectus* Parasitism Rate (Mean in %) (±SE)	% of Canola Fields above Damage Threshold
2015	21	1.80 (±0.37)	0.32 (±0.13)	2.29 (±0.93)	1.71 (±0.78)	48.39 (±11.54) ^2^	24.76 (±9.10) ^2^	4.76
2016	19	3.63 (±1.59)	3.45 (±1.48)	6.40 (±2.53)	4.00 (±1.71)	12.88 (±4.00)	5.40 (±2.01)	21.05
2017	20	7.15 (±2.88)	3.69 (±1.34)	3.33 (±1.36)	2.78 (±1.17)	9.81 (±2.54)	6.33 (±1.60)	35.00
2018	26	1.09 (±0.37)	0.86 (±0.26)	4.95 (±2.07)	3.53 (±1.64)	23.09 (±7.27)	14.93 (±5.32)	7.69
2019	28 ^1^	9.24 (±3.37)	6.91 (±2.15)	6.44 (±2.06)	4.88 (±1.52)	9.88 (±3.21)	6.10 (±2.37)	39.29
2020	26 ^1^	2.83 (±0.75)	6.43 (±1.66)	14.00 (±3.23)	10.72 (±2.52)	26.82 (±4.53)	21.76 (±3.95)	30.77

^1^ Five canola fields in Capitale-Nationale region, with a high cabbage seedpod weevil (CSW) abundance, were spatially close. ^2^ Some sampling sometimes showed a high parasitism rate but almost no emergence holes (CSW and parasitoids).

## Data Availability

The data used in this study would be made available by the corresponding author upon request.

## Supplementary Materials

The following supporting information can be downloaded at: https://www.mdpi.com/article/10.3390/insects14040327/s1, Figure S1: Relation between *T. perfectus* parasitism rate (mean in %) and CSW abundance (average number); Table S1: Final models representing the effects of landscape predictors on (i) CSW infestation rate (data were arcsine-square-root transformed) and on (ii) CSW abundance (data were log transformed); Table S2: Final model representing effects of landscape predictors on *T. perfectus* parasitism rate.
