# Supplementary material for: Landscape Effects on the Cabbage Seedpod Weevil, Ceutorhynchus obstrictus (Coleoptera: Curculionidae), and on Its Parasitoid, Trichomalus perfectus (Hymenoptera: Pteromalidae), in Canola
Source: Insects. 2023 Mar 28;14(4):327. doi: 10.3390/insects14040327 (PMC10145420; doi:10.3390/insects14040327)
Supplement: Supplementary file 1 [file insects-14-00327-s001.zip › insects-2277619-supplementary.pdf]

Supplementary Materials

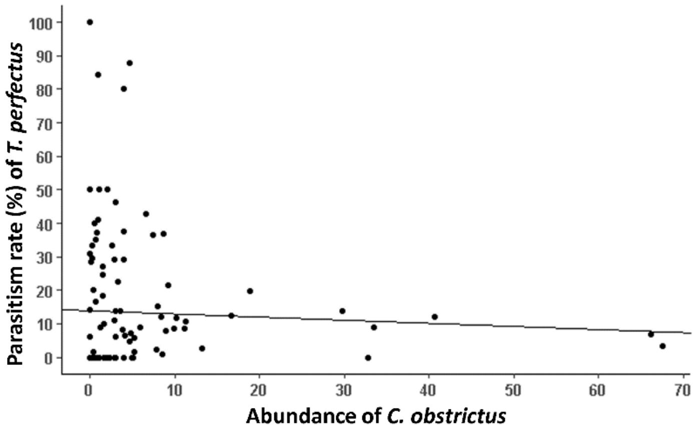

Figure S1. Relation between *T. perfectus* parasitism rate (mean in %) and CSW abundance (average number).

Table S1. Final models representing the effects of landscape predictors on (i) CSW infestation rate (data were arcsine-square-root transformed) and on (ii) CSW abundance (data were log transformed).

Dependent variable	Predictor	Estimate	df	t-value	CI	p-value
**CSW infestation**	(Intercept)	0.09	11.41	1.69	[- 0.01, 0.19]	0.091
	% of roads	1.20	125.62	3.69	[0.56, 1.84]	**<0.001**
	% of cereals	0.14	126.98	2.05	[0.01, 0.28]	**0.041**
Observations	139	σ^2	7.98			
Marginal R^2 / Conditional R^2	0.000 / 0.002	*ICC*	0.00			
**CSW abundance**	(Intercept)	0.66	8.76	2.20	[0.07, 1.25]	**0.029**
	% of roads	8.17	122.49	3.26	[3.21, 13.13]	**0.001**
	% of cereals	1.09	125.37	2.09	[0.06, 2.13]	**0.039**
Observations	134	σ^2	0.46			
Marginal R^2 / Conditional R^2	0.050 / 0.595	*ICC*	0.57			

Table S2. Final model representing effects of landscape predictors on *T. perfectus* parasitism rate.

Predictor	Estimate	SE	z-value	CI	p-value
(Intercept)	-3.88	0.62	-6.22	[0.01, 0.07]	**<0.001**
Hedgerow length	- 0.04	0.01	- 5.70	[0.95, 0.98]	**<0.001**
Distance to water	- 0.03	0.01	- 3.31	[0.96, 0.99]	**0.001**
% of roads	-5.22	1.52	-3.44	[0.00, 0.11]	**0.001**
Shannon index (total)	1.12	0.24	4.64	[1.91, 4.93]	**<0.001**
Average perimeter-to-area ratio of crops	3.13	0.84	3.71	[4.38, 119.51]	**<0.001**
% hay/pastures	1.47	0.46	3.23	[1.79, 10.69]	**0.001**
Abundance of CSW	0.00	0.00	0.63	[0.99, 1.01]	0.53
% of soybean	1.93	0.72	2.66	[1.66, 28.40]	**0.008**
Observations	112	σ^2	3.29		
Marginal R^2 / Conditional R^2	0.050 / 0.280	ICC	0.24		
